# Supplementary material for: Aberrantly Expressed Hsa_circ_0060762 and CSE1L as Potential Peripheral Blood Biomarkers for ALS
Source: Biomedicines. 2023 Apr 28;11(5):1316. doi: 10.3390/biomedicines11051316 (PMC10215595; doi:10.3390/biomedicines11051316)
Supplement: Supplementary file 1 [file biomedicines-11-01316-s001.zip › Ravnik-Glavac_Biomedicines_Suppl. Table S1.pdf]

Supp.Table S1. List of qPCR primers.

**Target RNA**	**Primer sequence (5′ – 3′)**
hsa_circ_0060762	F: CCAAAATTCACCTGGCACAG
	R: GGTGGTCATTTGCTTTGG
CSE1L	QuantiTect: Hs_CSE1L_1_SG (Cat. No. QT00015498)
RPS17	F: CCATTATCCCCAGCAAAAAG
	R: GAGACCTCAGGAACATAATTG
RPL13A	QuantiTect: Hs_RPL13A_1_SG (Cat. No. QT00089915)
