# Supplementary material for: Aberrantly Expressed Hsa_circ_0060762 and CSE1L as Potential Peripheral Blood Biomarkers for ALS
Source: Biomedicines. 2023 Apr 28;11(5):1316. doi: 10.3390/biomedicines11051316 (PMC10215595; doi:10.3390/biomedicines11051316)
Supplement: Supplementary file 1 [file biomedicines-11-01316-s001.zip › Ravnik-Glavac_Biomedicines_Suppl. Table S2.pdf]

Suppl. Table S2. Constraints metrics and regression statistics of genes with circRNA showing a FDR<0.1).

GENE_ID	GENE_ NAME	DSC_score	SSC_score	pLI	R2	p-value	FDR	p-value (Bonferroni)	circRNA
ENSG00000108510	***MED13***	-2.36	-2.51	1	0.35	0.005521	0.087	0.524	*hsa_circRNA_405614*
ENSG00000108424	***KPNB1***	-2.50	-2.61	1	0.35	0.005477	0.087	0.52	*hsa_circRNA_102111*
ENSG00000151461	***UPF2***	-2.20	-2.71	1	0.39	0.003135	0.074	0.297	*hsa_circRNA_007448*
ENSG00000082898	***XPO1***	-2.30	-2.84	1	0.40	0.002702	0.074	0.256	*hsa_circRNA_102732*
ENSG00000151461	***UPF2***	-2.20	-2.71	1	0.48	0.000621	0.029	0.059	*hsa_circRNA_100548*
ENSG00000124207	***CSE1L***	-2.42	-2.98	1	0.62	0.000034	0.003	0.003	*hsa_circRNA_060762*
