# Supplementary material for: Aberrantly Expressed Hsa_circ_0060762 and CSE1L as Potential Peripheral Blood Biomarkers for ALS
Source: Biomedicines. 2023 Apr 28;11(5):1316. doi: 10.3390/biomedicines11051316 (PMC10215595; doi:10.3390/biomedicines11051316)
Supplement: Supplementary file 1 [file biomedicines-11-01316-s001.zip › Ravnik-Glavac_Biomedicines_Suppl. Table S3.pdf]

Supp. Table S3. Correlations between circRNA and gene expression levels in ALS patients and association with clinical variables.

	Sex	ALS onset	Age at the time of blood collection	Age at onset	Disease duration
**Sex**	-	0.289*	0.176	0.146	-0.166
**ALS onset**		-	0.187	0.202	-0.294*
**Age at the time of blood collection**			-	0.991**	-0.037
**Age at onset**				-	-0.113
**Disease duration**					-
**Survival time**					
**Level of functional impairment**					
**Rate of progression**					
**CSE1L**					
**Has_circ_0060762**					

	Survival time	Level of functional impairment	Rate of progression	CSE1L	Has_circ_0060762
**Sex**	0.073	0.077	-0.165	-0.133	-0.051
**ALS onset**	-0.211	0.288*	-0.225	-0.098	0.194
**Age at the time of blood collection**	0.160	-0.177	-0.241	0.056	-0.032
**Age at onset**	0.107	-0.148	-0.307	0.072	-0.033
**Disease duration**	0.817**	-0.418**	0.593**	0.043	0.053
**Survival time**	-	-0.451*	0.652**	-0.173	0.162
**Level of functional impairment**		-	-0.177	0.116	0.147
**Rate of progression**			-	-0.108	0.026
**CSE1L**				-	0.289*
**Has_circ_0060762**					-
